# Clinical and Laboratory Profiles, Time to Presentation, and Antibacterial Therapy in Children with Aseptic Meningitis: A Comparison of Confirmed Enteroviral and Unidentified-Etiology Cases

**DOI:** 10.3390/epidemiologia7040115

**Published:** 2026-08-20

**Authors:** Khatuna Devdariani, Bakhyt Kosherova, Xeniya Mkhitaryan, Yelena Pozdnyakova, Aynash Dyusembaeva, Gulsharbat Alshynbekova

**Affiliations:** 1 Department of Infectious Diseases and Phthisiology, Karaganda Medical University, Karaganda 100008, Kazakhstan; devdariani@qmu.kz (K.D.);; 2 Office of the Rector, Karaganda Medical University, Karaganda 100008, Kazakhstan; 3 Department of Physiology, Karaganda Medical University, Karaganda 100008, Kazakhstan; 4 Department of Biomedicine, Karaganda Medical University, Karaganda 100008, Kazakhstan

**Keywords:** aseptic meningitis, enterovirus, children, cerebrospinal fluid, pediatric infectious diseases, meningitis epidemiology, antibacterial therapy, diagnostic stewardship, seasonality, unidentified etiology

## Abstract

Background/Objectives: Pediatric aseptic meningitis remains diagnostically challenging because early manifestations may be incomplete, cerebrospinal fluid (CSF) findings vary with disease stage, and etiologic confirmation is not always available. This study compared hospitalized children with PCR-confirmed enteroviral meningitis and meningitis of unidentified etiology, focusing on presentation timing, CSF patterns, and antibacterial therapy. Methods: This single-center retrospective observational study included 87 pediatric patients hospitalized with aseptic meningitis or meningoencephalitis. Patients were classified into a PCR-confirmed enteroviral group (EV+, *n* = 58) and an unidentified-etiology group (*n* = 29). Clinical characteristics, time from symptom onset to admission, meningeal signs, peripheral blood inflammatory markers, CSF parameters, associations among CSF variables, and antibacterial therapy were analyzed. Results: A marked summer pattern was observed, with 73 of 87 cases occurring from June to August. Most patients were admitted 2–5 days after symptom onset. Compared with the unidentified-etiology group, EV+ patients had lower body temperature, total leukocyte count, neutrophil percentage, and absolute neutrophil count, and a higher lymphocyte percentage. CSF cell count, protein, glucose, and chloride did not differ significantly between groups. In both groups, CSF cell count correlated positively with protein concentration. CSF glucose was unrelated to inflammatory CSF markers in the EV+ group but correlated negatively with CSF cell count and protein in the unidentified-etiology group. Antibacterial therapy was more frequent in the unidentified-etiology group. Conclusions: Combining clinical presentation, peripheral blood markers, CSF relationships, and molecular findings may improve diagnostic confidence and support more appropriate antibacterial treatment decisions in children with suspected aseptic meningitis.

## 1. Introduction

Acute inflammatory diseases of the central nervous system in children are among the most clinically challenging conditions encountered in infectious disease hospitals, as they require rapid decision-making in situations where diagnostic errors may have serious consequences [1,2]. Pediatric meningitis typically presents with a combination of nonspecific symptoms, including fever, headache, vomiting, and lethargy, while individual clinical signs have limited predictive value at the time of admission [3]. Even with modern diagnostic technologies, the early differentiation of bacterial meningitis from viral meningitis remains challenging because their clinical presentations may be similar and inflammatory laboratory markers may overlap between the two etiologic categories [4]. Consequently, empirical antibacterial therapy is commonly initiated in patients with suspected meningitis before microbiological results become available. Although this approach improves patient safety when bacterial meningitis cannot be excluded, it also increases the risk of unnecessary antibiotic exposure and may prolong hospitalization in patients with viral meningitis. This problem is a major focus of current diagnostic stewardship and antimicrobial stewardship strategies, which aim to accelerate pathogen identification and optimize antimicrobial therapy [5,6].

Enteroviruses are among the leading causes of aseptic meningitis in children worldwide. Their epidemiological importance is attributable to their high prevalence, extensive serotype diversity, and pronounced seasonal circulation [7,8,9]. In recent years, growing evidence has indicated that the epidemiology of viral meningitis and enterovirus infections is sensitive to changes in social behavior and public health interventions [10]. Studies conducted during the COVID-19 pandemic documented a marked decline in pediatric enteroviral meningitis in several regions in 2020, followed by a subsequent resurgence in enterovirus circulation. These findings highlight the dynamic nature of seasonal epidemiological patterns and the need for local hospital-based studies that reflect real-world diagnostic and hospitalization practices [11,12].

The conventional cerebrospinal fluid profile of viral meningitis includes lymphocytic pleocytosis, a moderate elevation in protein concentration, and generally preserved glucose levels [13]. However, in clinical practice, this pattern is subject to substantial variability. An important finding reported in recent years is that enterovirus RNA may be detected by PCR in cerebrospinal fluid even in the absence of pleocytosis, particularly in infants and when lumbar puncture is performed early in the course of illness [14]. This finding has direct clinical implications: confirmation of an enteroviral etiology in patients with atypical cerebrospinal fluid findings may allow clinicians to safely shorten the duration of antibiotic therapy and optimize patient management. In contrast, when rapid etiologic confirmation is unavailable, empirical antibacterial therapy is more likely to be continued [15]. Thus, an average cerebrospinal fluid profile alone is insufficient to guide clinical decision-making. Patient age, the timing of lumbar puncture relative to symptom onset, and the relationship between cerebrospinal fluid findings and systemic inflammatory markers in peripheral blood should also be considered.

Another important aspect of the problem is the high proportion of cases classified in routine hospital registries as being of unidentified etiology. In clinical practice, this category is heterogeneous and may include patients who were not tested, were tested late in the disease course, or underwent testing with a limited diagnostic panel, as well as patients with infections caused by other viruses or with noninfectious conditions that mimic aseptic meningitis [16]. Therefore, a comparison between PCR-confirmed enteroviral meningitis (EV+) and meningitis of unidentified etiology is methodologically justified. Such a comparison allows the identification of admission parameters that may contribute to etiologic differentiation and those whose diagnostic value is limited when the causative pathogen remains unidentified. Contemporary studies of diagnostic stewardship indicate that the implementation of rapid molecular diagnostic panels, including meningitis/encephalitis panels, can alter clinical management. However, substantial interhospital variability persists in test utilization and result interpretation, underscoring the importance of local data for the development of clinically relevant recommendations [5].

When the etiology remains uncertain, the selection of empirical therapy requires a careful balance between ensuring patient safety and avoiding unnecessary antibiotic use. In suspected bacterial meningitis, prompt administration of antibiotics is warranted; however, in viral meningitis, antibacterial therapy may be unnecessary. Timely etiologic confirmation, including through molecular diagnostic methods, is therefore important not only for establishing the diagnosis but also for limiting unwarranted antibiotic exposure. Accordingly, pediatric aseptic meningitis lies at the intersection of clinical diagnosis, diagnostic stewardship, and antimicrobial stewardship [17]. When bacterial meningitis is suspected, the selection of an antibacterial agent also depends on its pharmacokinetic properties, particularly its ability to penetrate the blood–brain and blood–cerebrospinal fluid barriers. Recent reviews indicate that carbapenems, including meropenem, may be appropriate for severe central nervous system infections when clinically indicated; however, meropenem exposure in cerebrospinal fluid is variable and is influenced by the degree of meningeal inflammation, the dosing regimen, and patient-specific pharmacokinetic factors [18,19]. Thus, accurate estimation of the likelihood of a viral etiology is not merely a diagnostic consideration but also an essential component of rational pharmacotherapy and the avoidance of unnecessary antibiotic exposure.

Beyond comparisons of individual cerebrospinal fluid parameters, increasing attention is being paid to diagnostic patterns and interrelationships among cerebrospinal fluid variables. From a clinical perspective, cerebrospinal fluid should be viewed as a set of interrelated parameters: pleocytosis reflects the cellular component of the inflammatory response; protein concentration reflects barrier dysfunction and exudation; and glucose concentration reflects metabolic processes influenced by transport across the blood–brain and blood–cerebrospinal fluid barriers, as well as by glucose consumption by inflammatory cells and microorganisms. Accordingly, the interpretation of cerebrospinal fluid findings in patients with suspected meningitis should not be based on any single marker in isolation but rather on an integrated assessment of pleocytosis, differential cell count, protein concentration, glucose concentration, and their clinical and laboratory interrelationships [20]. The association between pleocytosis and protein concentration may reflect the combined intensity of the cellular inflammatory response and barrier dysfunction: pleocytosis represents the cellular component of inflammation, whereas an elevated protein concentration is associated with increased permeability of the blood–brain and blood–cerebrospinal fluid barriers and with exudative changes. The contribution of glucose to the cerebrospinal fluid profile is more dependent on the etiology and stage of the disease: a decreased cerebrospinal fluid glucose concentration is more commonly associated with bacterial, fungal, or tuberculous meningitis, whereas glucose concentrations are usually preserved in viral meningitis [21]. The current diagnostic trend in central nervous system infections is away from reliance primarily on clinical and routine cerebrospinal fluid criteria and toward etiologically informed patient stratification. This shift is particularly evident in specific forms of meningitis, including tuberculous meningitis, for which molecular, immunologic, and protein biomarkers are under active investigation. The same principle applies to pediatric aseptic meningitis because clinical features and routine cerebrospinal fluid findings do not always reliably distinguish confirmed viral meningitis from cases in which no causative pathogen is identified [22].

The evaluation of relationships among cerebrospinal fluid parameters requires the use of appropriate statistical methods, particularly when data are non-normally distributed and subgroup sizes are relatively small. In this context, retrospective cohorts from infectious disease hospitals provide valuable real-world data, enabling the assessment of both differences in individual laboratory parameters and the consistency of associations among cerebrospinal fluid variables in routine clinical practice [23,24].

Therefore, it is important to determine which clinical and laboratory parameters available at admission differ between patients with PCR-confirmed enteroviral meningitis and those with aseptic meningitis of unidentified etiology. Of particular interest are not only differences in blood and cerebrospinal fluid parameters between the groups but also the associations among cerebrospinal fluid biomarkers within each group, as these patterns may reflect differences in the inflammatory response and underlying etiologic heterogeneity.

The aim of this study was to compare the clinical and laboratory profiles of pediatric patients with PCR-confirmed enteroviral meningitis (EV+) and those with aseptic meningitis of unidentified etiology and to evaluate the strength and direction of the associations among cerebrospinal fluid cell count, protein concentration, and glucose concentration within each group using retrospectively collected data from a pediatric infectious disease hospital.

## 2. Materials and Methods

### 2.1. Study Design and Setting

This was a single-center retrospective observational study conducted in the pediatric infectious disease department of the Regional Infectious Diseases Hospital, Karaganda, Kazakhstan. The analysis included data from patients hospitalized with a clinical diagnosis of aseptic meningitis/meningoencephalitis during the 2024–2025 study period. Routinely collected clinical and laboratory data were extracted from medical records.

### 2.2. Study Participants

Hospital electronic medical records were screened for all pediatric admissions with a diagnosis of aseptic meningitis or aseptic meningoencephalitis during the study period. After applying the eligibility criteria, 87 patients were included in the final analysis. Patient identifiers and admission dates were reviewed, and no recurrent admissions of the same patient were identified; therefore, each record represented one unique patient and one hospitalization episode.

### 2.3. Eligibility Criteria

#### 2.3.1. Inclusion Criteria

Patients were included if all of the following criteria were met:

1. Age younger than 18 years;
2. Hospitalization in the pediatric infectious disease department during the 2024–2025 study period;
3. A clinical diagnosis of aseptic meningitis or aseptic meningoencephalitis documented in the medical record;
4. Lumbar puncture performed during hospitalization, with cerebrospinal fluid findings consistent with non-purulent inflammatory involvement of the central nervous system;
5. Availability of the clinical, laboratory, and etiological information required to confirm eligibility and assign the patient to either the enterovirus-positive group or the unidentified etiology group.

#### 2.3.2. Exclusion Criteria

Patients were excluded if they had confirmed tuberculous meningitis, HSV-1/2 PCR-positive infection, another identified non-enteroviral infectious etiology, or a non-infectious condition inconsistent with the diagnosis of aseptic meningitis or aseptic meningoencephalitis. Medical records were also excluded if the available information was insufficient to verify the diagnosis, determine etiological group assignment, or extract the clinical and laboratory data required for the analysis. Patient identifiers and admission dates were reviewed for recurrent admissions. No recurrent admissions of the same patient were identified during the study period; therefore, each included record represented one unique patient and one hospitalization episode.

### 2.4. Group Formation and Etiological Definition

Enteroviral (EV+) etiology was defined as the detection of enterovirus RNA in cerebrospinal fluid by qualitative real-time reverse-transcription polymerase chain reaction (RT-PCR). Testing was performed as part of routine clinical diagnostics using commercially available assays for enterovirus RNA detection in accordance with the manufacturers’ instructions. Amplification and fluorescence detection were carried out using the Rotor-Gene Q real-time PCR system (QIAGEN, Hilden, Germany). A result was considered positive when a specific enterovirus amplification signal was detected according to the assay interpretation criteria and all internal control requirements were met.

The EV+ group comprised patients with enterovirus RNA detected in cerebrospinal fluid by RT-PCR (*n* = 58). All patients assigned to the unidentified etiology group underwent enterovirus RT-PCR testing of cerebrospinal fluid and had negative results (*n* = 29). No alternative causative pathogen was identified in these patients during the diagnostic evaluation documented in their medical records.

### 2.5. Variables

The analyzed variables included clinical parameters at admission, peripheral blood parameters, and cerebrospinal fluid parameters. Clinical data included body temperature at admission, time from disease onset to hospitalization, and the presence of headache, vomiting, meningeal signs, and seizures when these were documented in the medical record. Blood parameters included total leukocyte count, the percentages of neutrophils and lymphocytes, and the absolute neutrophil count (ANC), calculated using the following formula: ANC (cells/µL) = leukocyte count (×10^9^/L) × 1000 × neutrophils (%)/100. Cerebrospinal fluid parameters included cell count, protein, glucose, and chloride levels. The analyses were based on the initial diagnostic CSF sample obtained during hospitalization.

### 2.6. Statistical Analysis

Statistical analysis was performed using GraphPad Prism 8.0.1. The distribution of each continuous variable was assessed separately within the study groups using the Shapiro–Wilk test. Because several variables showed statistically significant deviations from a normal distribution, continuous data were analyzed using non-parametric methods and are presented as median and interquartile range [Q1; Q3]. Comparisons between the EV+ and unidentified etiology groups were performed using the Mann–Whitney U test for continuous variables and the χ^2^ test or Fisher’s exact test for categorical variables, depending on expected cell frequencies. Within-group relationships among cerebrospinal fluid parameters were assessed using Spearman’s rank correlation coefficient (ρ). All tests were two-sided, and *p* < 0.05 was considered statistically significant.

To assess the association between time to presentation and clinical parameters, patients were additionally stratified according to the day of admission from disease onset. For the analysis of early meningeal signs, patients admitted on the first day of illness were compared with those admitted on day 2 or later; the frequency of clearly positive nuchal rigidity was evaluated using Fisher’s exact test. For the analysis of antibacterial therapy, only the two main analytical groups were compared: patients with confirmed enteroviral etiology and patients with unidentified etiology. Patients with specific non-enteroviral etiologies, including HSV-1/2 PCR-positive cases, were not included in the main comparative analysis. Odds ratios (ORs) were calculated with 95% confidence intervals.

## 3. Results

### 3.1. Demographic Characteristics of the Study Cohort

The final study cohort included 87 pediatric patients. Of these, 57 were male (65.52%) and 30 were female (34.48%). The largest age groups were children aged 10–14 years (37.93%) and 5–9 years (31.03%), whereas only one patient (1.15%) was younger than 1 year (Table 1).

### 3.2. Time from Disease Onset to Hospital Admission and Clinical Severity

To assess the temporal profile of hospitalization, the time from the onset of clinical symptoms to hospital admission was analyzed. The distribution of patients by time to presentation was uneven: most children were admitted not on the first day of illness, but between days 2 and 5 after symptom onset.

Thirteen patients were hospitalized on the first day of illness, accounting for 14.9% of the total cohort. Twenty-five patients (28.7%) were admitted on day 2, 17 patients (19.5%) on day 3, and 21 patients (24.1%) on days 4–5. Late presentation, defined as admission on day 6 of illness or later, was observed in 11 patients (12.6%). Thus, 63 of 87 patients, or 72.4%, were hospitalized between days 2 and 5 of illness.

To assess the association between time to presentation and disease severity, the following indicators were used: severe disease, transfer to the intensive care unit (ICU), length of hospital stay, and cerebrospinal fluid (CSF) cell count. Severe disease was uncommon and was observed in only 2 of 87 patients (2.3%). Transfer to the ICU was also recorded in 2 patients (2.3%). No clear increase in the frequency of severe disease or ICU transfer was observed among patients who presented later (Table 2).

The median length of hospital stay was comparable across all groups stratified by time to presentation: 9 days among patients admitted on day 1, 9 days on day 2, 8 days on day 3, 10 days on days 4–5, and 9 days among those admitted on day 6 or later. This indicates that later presentation was not associated with a longer hospital stay.

At the same time, the intensity of the cellular inflammatory response in the CSF tended to be higher among patients who presented later. The median CSF cell count increased from 34 [25; 71] cells/µL in patients admitted on the first day of illness to 159 [48.5; 248] cells/µL in patients admitted on days 4–5 and 145 [46.5; 542.5] cells/µL in those admitted on day 6 or later. Therefore, the time from disease onset to admission should be taken into account when interpreting CSF parameters, as later presentation may reflect a more developed stage of the cellular inflammatory response in the CSF.

Thus, admission between days 2 and 5 from disease onset predominated in the study cohort. Descriptively, later presentation was not accompanied by a clear increase in length of hospital stay, severe disease, or ICU transfer. However, median CSF cell count was descriptively higher among patients admitted after the first day of illness, suggesting that the time from disease onset may be relevant when interpreting CSF findings.

### 3.3. Timing and Frequency of Lumbar Puncture

In the vast majority of cases, lumbar puncture was performed early during hospitalization. In 91.95% of patients, cerebrospinal fluid was obtained immediately upon admission to the hospital, and in an additional 1.15% it was obtained within the first 24 h. Delayed lumbar puncture, performed on days 2–3 or later, was recorded in only 6.9% of cases. This indicates that the cerebrospinal fluid parameters in the study cohort predominantly reflected the patient’s condition at the stage of initial in-hospital diagnostic evaluation.

In most cases, only one diagnostic lumbar puncture was performed. A single lumbar puncture was performed in 95.4% of patients, whereas repeat puncture was required in 4 patients (4.6%); one patient underwent a series of three lumbar punctures. These data show that repeat cerebrospinal fluid monitoring was rarely required in the study cohort.

### 3.4. Seasonal Distribution of Hospitalizations

The monthly distribution of hospitalizations was markedly uneven. Cases of aseptic meningitis were registered predominantly during the warm season, whereas the number of cases was substantially lower during the winter and spring months. The most pronounced increase in hospitalizations was observed from June to August, with the peak number of admissions recorded in July (Table 3).

Thus, most hospitalizations occurred during the summer months: June, July, and August accounted for 73 of 87 cases (83.91%). The highest number of hospitalizations was recorded in July, with 32 cases (36.78%). In contrast, only 5 cases (5.75%) were registered in January and February. These findings indicate a pronounced seasonal concentration of aseptic meningitis cases in the study cohort.

### 3.5. Admission Temperature and Blood Inflammatory Profile

**Figure 1:**
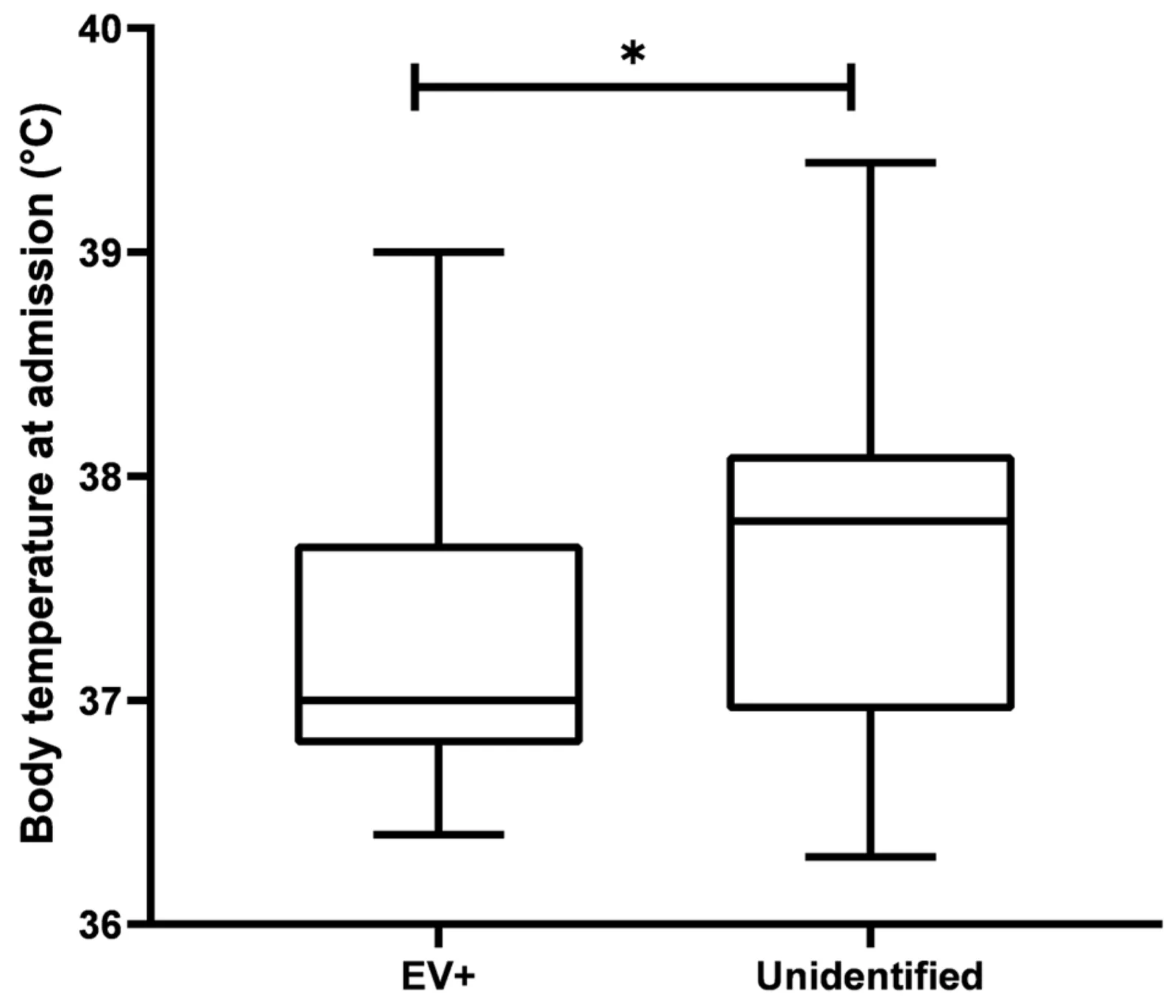
Admission body temperature in children with confirmed enteroviral aseptic meningitis and aseptic meningitis of unidentified etiology. Data are presented as median with interquartile range and whiskers. p values were calculated using the Mann–Whitney U test. * *p* < 0.05.

Body temperature at admission was significantly lower in patients with confirmed enteroviral etiology (EV+) than in patients with unidentified etiology: 37.00 [36.80; 37.70] °C and 37.80 [36.95; 38.10] °C, respectively; Mann–Whitney test, two-sided exact *p* = 0.0305. This indicates a less pronounced febrile response in patients with confirmed enteroviral etiology at the time of hospitalization (Figure 1).

**Figure 2:**
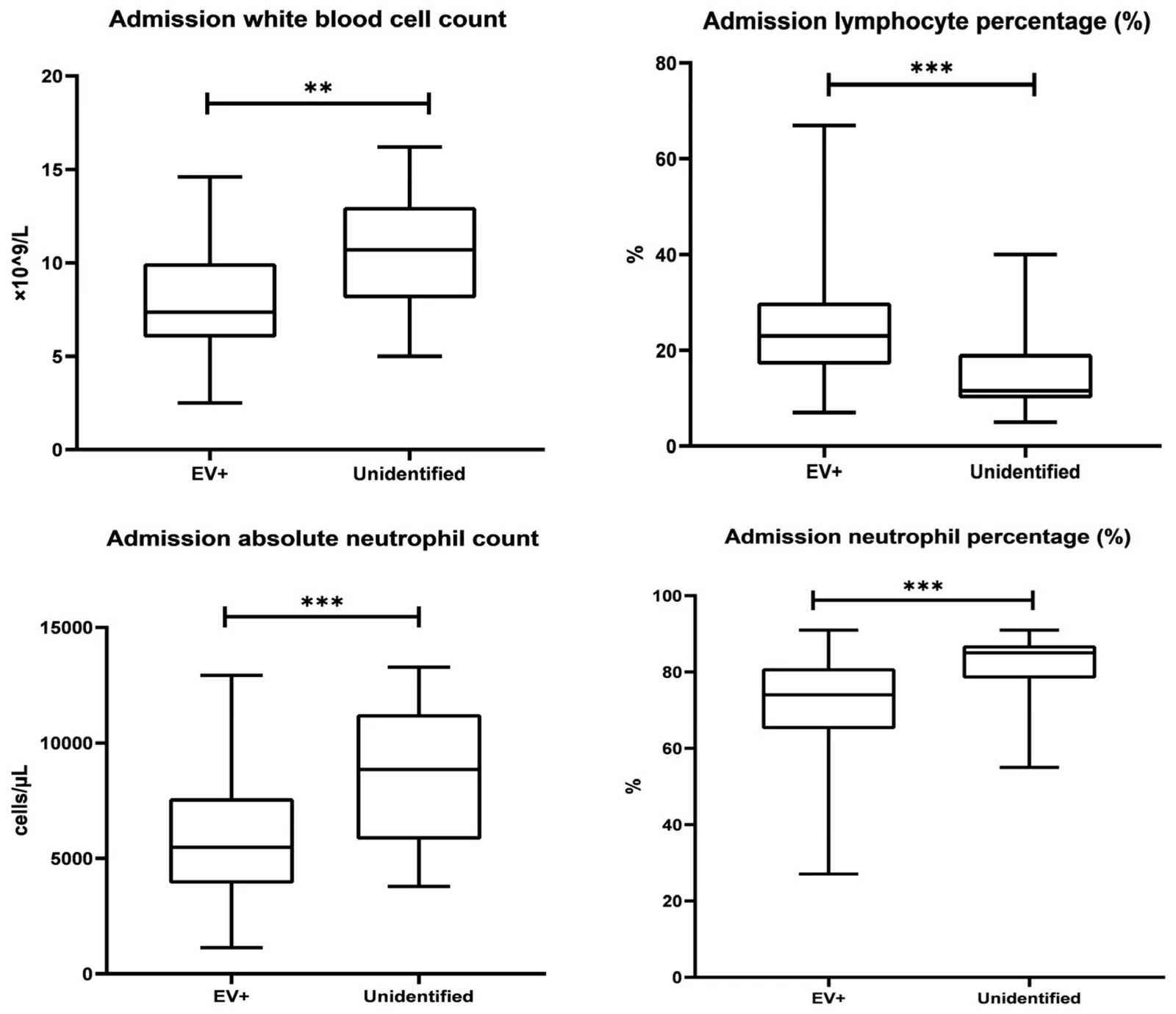
Blood inflammatory response parameters in children with confirmed enteroviral aseptic meningitis and aseptic meningitis of unidentified etiology. Data are presented as median with interquartile range and whiskers. *p* values were calculated using the Mann–Whitney U test. ** *p* < 0.01; *** *p* < 0.001.

The blood leukocyte count at admission was significantly lower in patients with confirmed enteroviral etiology (EV+) than in patients with unidentified etiology: 7.35 [6.00; 9.98] × 10^9^/L and 10.70 [8.10; 13.00] × 10^9^/L, respectively; Mann–Whitney test, two-sided exact *p* = 0.0014. This indicates a less pronounced peripheral blood inflammatory profile in the EV+ group. (Figure 2).

The proportion of neutrophils in peripheral blood at admission was also significantly lower in patients with confirmed enteroviral etiology (EV+) than in patients with unidentified etiology: 74.00 [65.00; 81.00]% and 85.00 [78.25; 87.00]%, respectively; Mann–Whitney test, two-sided exact *p* < 0.0001. This indicates a less pronounced neutrophilic component of the systemic inflammatory response in the EV+ group (Table 4).

The proportion of lymphocytes in peripheral blood at admission was significantly higher in patients with confirmed enteroviral etiology (EV+) than in patients with unidentified etiology: 23.00 [17.00; 30.00]% and 11.50 [10.00; 19.25]%, respectively; Mann–Whitney test, two-sided exact *p* < 0.0001. This indicates a higher relative lymphocyte proportion in the EV+ group.

The absolute neutrophil count (ANC) at admission was significantly lower in patients with confirmed enteroviral etiology (EV+) than in patients with unidentified etiology: 5476 [3905; 7603] and 8849 [5812; 11,247] cells/µL, respectively; Mann–Whitney test, two-sided exact *p* = 0.0002. This further reflects a less pronounced neutrophilic component of the systemic inflammatory response in the EV+ group.

### 3.6. Cerebrospinal Fluid Parameters

The cerebrospinal fluid (CSF) cell count at admission did not differ significantly between patients with confirmed enteroviral etiology (EV+) and those with unidentified etiology: 92.00 [39.00; 290.0] and 92.00 [26.00; 248.0] cells/µL, respectively; Mann–Whitney test, two-sided exact *p* = 0.5261. This indicates a comparable intensity of the cellular inflammatory response in the CSF in both groups at the time of hospitalization (Table 5).

**Figure 3:**
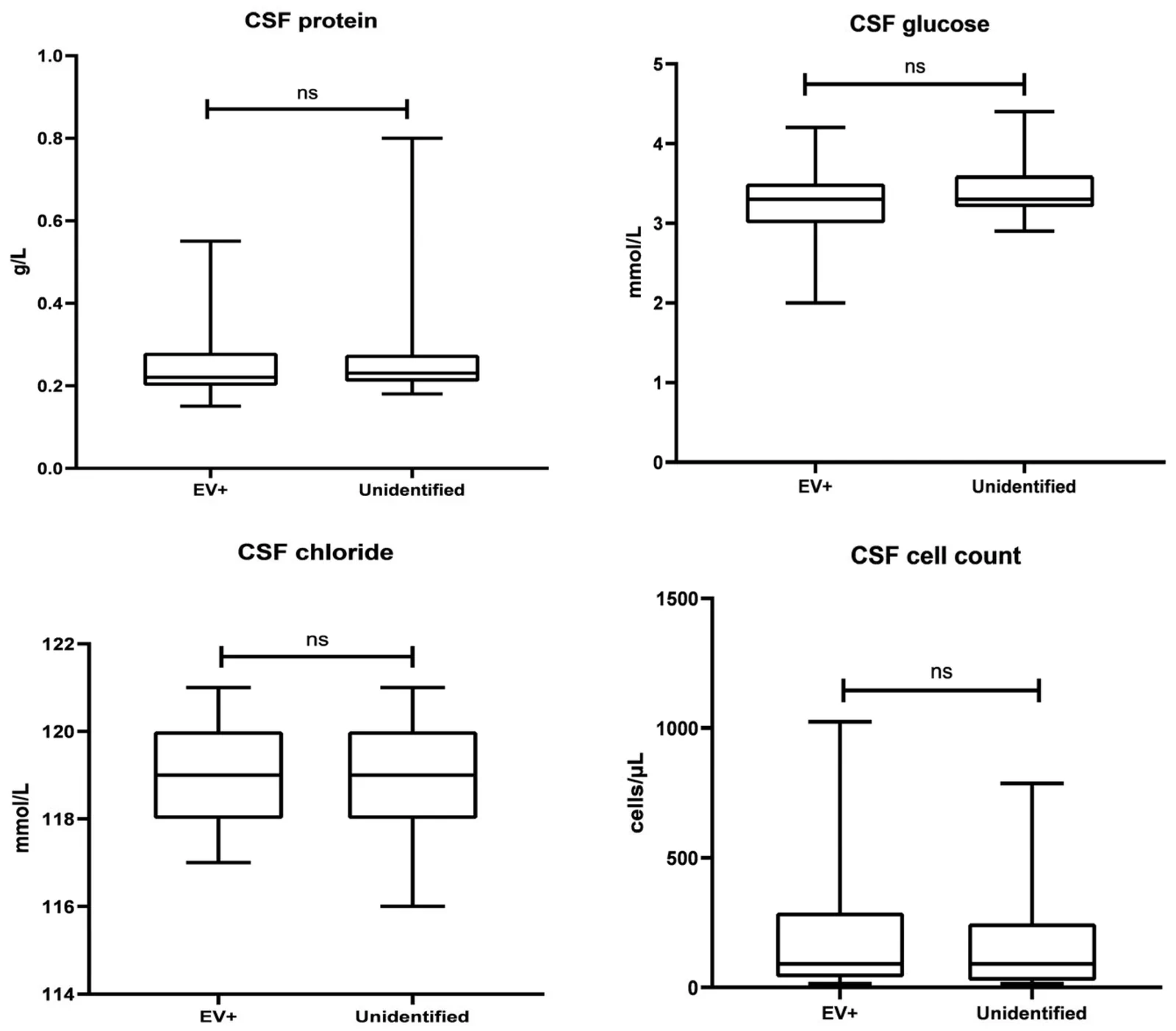
Cerebrospinal fluid parameters at admission according to etiological group. Data are presented as median with interquartile range and whiskers. *p* values were calculated using the Mann–Whitney U test. ns, not significant (*p* ≥ 0.05).

CSF protein at admission did not differ significantly between patients with confirmed enteroviral etiology (EV+) and those with unidentified etiology: 0.22 [0.20; 0.28] and 0.23 [0.21; 0.275] g/L, respectively; Mann–Whitney test, two-sided exact *p* = 0.4921. This indicates the absence of statistically significant between-group differences in CSF protein levels at the time of hospitalization (Figure 3).

CSF glucose at admission did not differ significantly between patients with confirmed enteroviral etiology (EV+) and those with unidentified etiology: 3.30 [3.00; 3.50] and 3.30 [3.20; 3.60] mmol/L, respectively; Mann–Whitney test, two-sided exact *p* = 0.2750. This indicates comparable CSF glucose levels in both groups at the time of hospitalization.

CSF chloride levels at admission did not differ significantly between patients with confirmed enteroviral etiology (EV+) and those with unidentified etiology: 119.0 [118.0; 120.0] and 119.0 [118.0; 120.0] mmol/L, respectively; Mann–Whitney test, two-sided exact *p* = 0.6220. This indicates comparable CSF chloride levels in both groups at the time of hospitalization.

### 3.7. Within-System Relationships Among Cerebrospinal Fluid Parameters

Spearman’s correlation analysis was used to assess relationships among CSF cell count, protein, glucose, and chloride within each etiological group (Table 6 and Table 7).

In the EV+ group, the Spearman correlation matrix showed a pronounced positive correlation between CSF cell count and CSF protein (ρ = 0.710; *p* < 0.0001), indicating coupling between the cellular inflammatory response and the protein component of CSF changes. Correlations between CSF cell count and glucose (ρ = −0.030; *p* = 0.8354), CSF cell count and chloride (ρ = −0.034; *p* = 0.8081), CSF protein and glucose (ρ = −0.001; *p* = 0.9937), and CSF protein and chloride (ρ = −0.089; *p* = 0.5282) were not statistically significant. A moderate positive correlation was observed between CSF glucose and chloride (ρ = 0.341; *p* = 0.0134); however, this relationship was not associated with the main inflammatory CSF markers, namely CSF cell count and protein.

In the unidentified etiology group, a positive relationship between CSF cell count and CSF protein was also observed (ρ = 0.619; *p* = 0.0003). In contrast to the EV+ group, CSF glucose demonstrated statistically significant negative correlations with inflammatory CSF parameters: with CSF cell count (ρ = −0.373; *p* = 0.0461) and with CSF protein (ρ = −0.371; *p* = 0.0478). This indicates a tendency toward lower CSF glucose values with greater expression of the cellular and protein components of CSF inflammation. Correlations between CSF chloride and CSF cell count (ρ = −0.324; *p* = 0.0861), CSF protein (ρ = −0.265; *p* = 0.1643), and CSF glucose (ρ = 0.317; *p* = 0.0933) did not reach statistical significance (Table 7).

Thus, in both etiological groups, the most consistent relationship was the positive correlation between CSF cell count and CSF protein. However, the role of glucose differed between groups: in confirmed enteroviral etiology, CSF glucose was virtually unrelated to CSF cell count and protein, whereas in the unidentified etiology group it showed weak to moderate but statistically significant negative correlations with both inflammatory parameters. Thus, the pattern of associations involving CSF glucose differed between the two etiological groups.

### 3.8. Nuchal Rigidity According to Time from Disease Onset to Admission Association Between Time to Presentation and Nuchal Rigidity

To assess the association between time to presentation and the severity of meningeal signs, an additional analysis was performed comparing the frequency of clearly positive nuchal rigidity in patients admitted on the first day of illness with that in patients admitted on day 2 or later.

Clearly positive nuchal rigidity on the first day of illness was recorded in 5 of 13 patients, accounting for 38.5%. In the remaining 8 patients admitted on the first day, nuchal rigidity was equivocal or absent. Among patients admitted on day 2 or later, positive nuchal rigidity was observed in 42 of 74 patients, or 56.8%; in 32 patients, or 43.2%, nuchal rigidity was equivocal or absent (Table 8).

Although the frequency of clearly positive nuchal rigidity was lower among patients admitted on the first day of illness, the difference between groups did not reach statistical significance: Fisher’s exact test, *p* = 0.2445; OR = 0.48; 95% CI: 0.16–1.45. Therefore, in this cohort, no statistically significant association was found between early presentation and the frequency of positive nuchal rigidity (Table 8).

At the same time, it is clinically important that in 61.5% of patients admitted on the first day of illness, nuchal rigidity was equivocal or absent. This indicates that the absence of clearly positive nuchal rigidity in the early stages of disease does not exclude aseptic meningitis and should not reduce diagnostic vigilance in the presence of fever, headache, vomiting, or general clinical deterioration.

### 3.9. Antibacterial Therapy According to Etiology

To assess the association between etiological status and the use of antibacterial therapy, the frequency of antibiotic administration was analyzed in patients with confirmed enteroviral etiology and in those with unidentified etiology.

Antibacterial therapy was administered to 15 of 58 patients with confirmed enteroviral meningitis, accounting for 26.3%. In the unidentified etiology group, antibiotics were administered to 15 of 29 patients, or 51.7%. Thus, antibacterial therapy was prescribed almost twice as often in patients with unidentified etiology as in those in the EV+ group (Table 9).

The difference between groups was statistically significant according to Fisher’s exact test (*p* = 0.0305). When the odds ratio was calculated for the EV+ group relative to the unidentified etiology group, the OR was 0.33 (95% CI: 0.13–0.82). This indicates that confirmed enteroviral etiology was associated with a lower likelihood of receiving antibacterial therapy. In the reciprocal interpretation, the odds of antibiotic administration in patients with unidentified etiology were approximately three times higher than in patients with confirmed enteroviral etiology: reciprocal OR = 3.00 (95% CI: 1.22–7.79).

Therefore, the absence of etiological verification was associated with more frequent use of antibacterial therapy. This finding may reflect clinical uncertainty and a more cautious management strategy in patients in whom the viral nature of the disease had not been laboratory-confirmed.

## 4. Discussion

The present study provides a detailed clinical and laboratory characterization of pediatric aseptic meningitis according to enterovirus RT-PCR status, time from disease onset to hospital admission, relationships among CSF parameters, and antibacterial treatment. The principal between-group differences were observed in the peripheral inflammatory response. Children with PCR-confirmed enteroviral meningitis had lower body temperature, total leukocyte count, neutrophil percentage, and absolute neutrophil count, together with a higher lymphocyte percentage, compared with patients in whom enterovirus RT-PCR was negative and no alternative causative pathogen was identified. In contrast, routine CSF parameters did not clearly differentiate between the etiologic groups. These findings indicate that peripheral blood parameters differentiated the study groups more clearly than the absolute values of routine CSF indices.

The timing of hospital presentation is important for interpreting both clinical and laboratory findings. Most patients were admitted between days 2 and 5 after symptom onset, whereas presentation on the first day was less common. Consequently, lumbar puncture was performed at different stages of the inflammatory process. Previous studies have shown that enterovirus may be detected in CSF even before pleocytosis develops, particularly when lumbar puncture is performed early in the disease course [15]. The interval between symptom onset and CSF sampling should therefore be considered when evaluating the intensity and cellular composition of the inflammatory response.

In the present cohort, a descriptively higher median CSF cell count was observed among children admitted on day 2 or later than among those admitted on the first day of illness. However, later presentation was not associated with a statistically significant increase in hospitalization duration, severe disease, or ICU transfer. This descriptive pattern may reflect the temporal evolution of meningeal inflammation rather than a proportional increase in clinical severity. Time to presentation should therefore be considered when interpreting CSF findings, although it was not associated with an unfavorable clinical course in this cohort.

The marked seasonal clustering of hospitalizations provides an epidemiological context for these findings. Most cases occurred during the summer months, corresponding to the recognized seasonality of enteroviral and other forms of viral meningitis. Population-based data from Kazakhstan have demonstrated a substantial burden of etiologically unspecified meningitis and an association between disease incidence and seasonal and climatic factors [25]. The current hospital-based observations complement national epidemiological evidence by showing how seasonal disease activity is reflected in pediatric admissions, presentation timing, laboratory findings, and treatment decisions.

All patients assigned to the unidentified etiology group underwent enterovirus RT-PCR testing of CSF and had negative results. Thus, this group represents enterovirus-negative aseptic meningitis without another identified causative pathogen rather than patients who were not examined for enterovirus. Nevertheless, a negative enterovirus result does not establish a single alternative diagnosis. Aseptic meningitis can be caused by multiple infectious agents, and other viral pathogens may produce a similar clinical and CSF profile. Varicella-zoster virus, for example, may cause meningitis and other neurological complications even in the absence of a typical cutaneous presentation [26].

The comparison between confirmed enteroviral meningitis and enterovirus-negative meningitis without an identified pathogen reflects two clinically relevant inpatient scenarios. In the first, a specific viral etiology is molecularly confirmed. In the second, the clinical and CSF findings support aseptic meningitis, but the causative agent remains unidentified. The differences observed between the groups may reflect the combined contribution of non-enteroviral etiologies, variation in the stage of inflammation, previous treatment, or other clinical factors not fully captured in retrospective medical records.

The analysis of nuchal rigidity illustrates the limitations of relying on an isolated physical sign. Clearly positive nuchal rigidity was documented in fewer than half of the children admitted on the first day of illness, and in 61.5% of early presenters this sign was equivocal or absent. Although the difference between the timing-of-admission groups did not reach statistical significance, the finding is clinically relevant. Pediatric evidence indicates that classic meningeal signs have limited sensitivity and cannot independently exclude meningitis [27]. Their expression may vary according to age, disease stage, inflammatory intensity, and examination conditions. Therefore, the absence of clearly positive nuchal rigidity early in the disease should not preclude lumbar puncture or further investigation when fever, headache, vomiting, clinical deterioration, or other compatible manifestations are present.

The peripheral blood profile of the EV+ group was more consistent with the inflammatory pattern generally associated with viral infection. Compared with the unidentified etiology group, these patients had lower leukocyte and neutrophil indices and a higher lymphocyte percentage. However, these variables are not etiologically specific. Viral CNS infections may be accompanied by systemic neutrophilia, and neutrophilic pleocytosis can occur during the early stages of enteroviral meningitis [28]. Thus, neither a neutrophilic peripheral blood response nor an early neutrophilic CSF pattern is sufficient to exclude enteroviral infection.

The observed hematological differences represent supportive findings that may contribute to early clinical assessment. Peripheral blood indices may also be influenced by presentation timing, previous therapy, age, host inflammatory response, and concurrent conditions. Current diagnostic approaches therefore emphasize combined assessment of clinical manifestations, blood parameters, CSF findings, and microbiological results rather than reliance on a single physical or laboratory marker [29].

In this cohort, peripheral blood variables differentiated the study groups more clearly than routine CSF indices. This does not diminish the diagnostic importance of lumbar puncture. CSF examination remained essential for demonstrating CNS inflammation, characterizing the general inflammatory pattern, and identifying findings suggestive of purulent or other specific forms of meningitis. The absence of significant differences in median CSF cell count, protein, glucose, and chloride indicates that these routine measurements were insufficient for distinguishing confirmed enteroviral disease from enterovirus-negative aseptic meningitis in the present sample.

Contemporary molecular diagnostic approaches can improve pathogen identification, but their yield depends on the timing of sampling, type of biological material, range of molecular targets included in the assay, analytical sensitivity, and use of additional typing or sequencing methods where appropriate [30]. In the present study, qualitative real-time RT-PCR provided direct confirmation of enterovirus RNA in CSF. Its clinical contribution was not to replace routine CSF examination, but to provide a specific etiological result that could be evaluated together with the clinical and laboratory profile.

CSF chloride demonstrated little variation and did not differ meaningfully between the etiological groups. It was also not consistently related to the principal indicators of CSF inflammation. These findings support its limited role in etiological differentiation. Current laboratory assessment places greater emphasis on CSF cell count, protein, glucose, microbiological testing, and molecular pathogen detection [30]. Chloride may remain part of a descriptive biochemical profile, but it contributed little to group discrimination in this cohort.

Analysis of relationships among CSF variables provided information beyond comparisons of their median values. The most consistent finding was the positive correlation between CSF cell count and protein concentration in both groups. Pleocytosis reflects inflammatory cell recruitment into the CSF, whereas elevated protein concentrations may accompany increased permeability or disruption of the blood–brain and blood–CSF barriers. Their parallel increase may therefore reflect a biologically plausible coordinated cellular and barrier response.

Because children admitted later had higher CSF cell counts, the cell count–protein relationship may partly reflect the temporal development of inflammation. Higher pleocytosis and protein concentrations were not accompanied by greater clinical severity, ICU transfer, or longer hospitalization. Clinical reviews of enteroviral meningitis similarly emphasize that lumbar puncture timing and the stage of the CSF inflammatory response should be considered when laboratory findings are used to guide observation and treatment [31].

CSF glucose showed a different pattern. In the EV+ group, glucose was not significantly associated with CSF cell count or protein, whereas in the unidentified etiology group it was negatively correlated with both inflammatory indicators. These correlations were modest, and no formal comparison of correlation coefficients between the groups was performed; therefore, they should be considered exploratory rather than evidence of a distinct pathophysiological mechanism.

Nevertheless, these findings illustrate the potential value of examining relationships among CSF parameters rather than assessing each measurement only in isolation. Research on CNS infections has shown that pathogen-related host responses may be reflected in multidimensional CSF biomarker and proteomic profiles that are not evident from individual marker concentrations [32]. Studies of pediatric tuberculous meningitis have likewise demonstrated that combinations of host CSF proteins may provide greater diagnostic information than isolated biomarkers [33]. Although the present analysis was limited to routine CSF indices, it supports further prospective investigation of coordinated CSF responses in pediatric meningitis.

Antibacterial therapy was administered significantly more frequently in the unidentified etiology group than in the EV+ group. The odds of receiving antibacterial treatment were approximately three times higher among patients in whom enterovirus RT-PCR was negative and no alternative pathogen was identified. This association is clinically plausible because the absence of a confirmed viral diagnosis leaves greater uncertainty regarding bacterial or other treatable causes. In contrast, molecular confirmation of enterovirus provides a specific etiologic explanation for the clinical presentation of aseptic meningitis and may support earlier reassessment of empirical antibacterial therapy.

Previous clinical guidance has indicated that timely confirmation of enterovirus may reduce unnecessary antibacterial exposure and assist decisions concerning treatment duration and hospitalization [31]. However, the present retrospective data cannot demonstrate that molecular confirmation itself caused the lower frequency of antibiotic use. Treatment decisions may also have been influenced by peripheral neutrophilia, body temperature, general clinical condition, presentation timing, previous treatment, physician judgment, and other variables not fully controlled in the analysis.

The present single-center cohort contributes clinically relevant information by linking seasonal admissions with presentation timing, early meningeal signs, peripheral inflammatory findings, CSF characteristics, etiological verification, and antibacterial therapy. The value of the study lies not in proposing a single marker of enteroviral infection, but in identifying which routinely available parameters differed according to etiologic status and which findings mainly reflected the presence or stage of CNS inflammation.

Overall, the principal distinctions between PCR-confirmed enteroviral meningitis and enterovirus-negative aseptic meningitis without another identified pathogen were observed in the peripheral inflammatory profile and the frequency of antibacterial therapy. Routine CSF values characterized meningeal inflammation but did not provide clear etiological separation. Within-group analysis demonstrated a consistent relationship between CSF cell count and protein and an exploratory difference in the pattern of associations involving CSF glucose. Pleocytosis and the expression of nuchal rigidity varied descriptively according to presentation timing, emphasizing the importance of evaluating clinical and laboratory findings in relation to the stage of illness.

## 5. Conclusions

This study demonstrated that pediatric aseptic meningitis was characterized by marked summer seasonality and predominant hospital admission between days 2 and 5 of illness. PCR-confirmed enteroviral meningitis differed from enterovirus-negative cases without an identified pathogen primarily by a less pronounced neutrophilic peripheral blood response, including lower total leukocyte count, neutrophil percentage, and absolute neutrophil count, together with a higher lymphocyte percentage.

Routine cerebrospinal fluid parameters at admission did not significantly distinguish between the etiological groups and varied in relation to disease timing. Within both groups, cerebrospinal fluid cell count was positively associated with protein concentration, whereas glucose showed no significant association with inflammatory parameters in the enterovirus-positive group and negative associations in the unidentified etiology group.

Antibacterial therapy was used more frequently in patients without confirmed enteroviral etiology. Overall, the study groups differed primarily in their peripheral blood inflammatory profiles and frequency of antibacterial therapy, whereas routine cerebrospinal fluid indices provided limited etiological discrimination and should be interpreted in relation to the timing of presentation.

## 6. Limitations

This study has several limitations. First, the sample size was relatively limited, particularly for the analysis of uncommon outcomes such as severe disease and ICU transfer. This may have reduced the statistical power to detect differences in these outcomes.

Second, the study was conducted at a single pediatric infectious diseases center. However, this hospital is the only specialized center in the city that admits children with suspected or confirmed infectious diseases of the central nervous system. Therefore, the study cohort is expected to represent the majority of hospitalized pediatric aseptic meningitis cases in the local population. Nevertheless, confirmation of the findings in multicenter cohorts from other regions would be valuable.

Third, all patients in the unidentified etiology group underwent enterovirus RT-PCR testing of cerebrospinal fluid and had negative results. However, a uniform comprehensive panel covering all potential viral and non-viral causes of aseptic meningitis was not available for the entire cohort. Consequently, the specific causative pathogen could not be identified in this group.

Fourth, the analysis was based primarily on the initial CSF examination performed during hospitalization. Repeat lumbar puncture was performed in only a small number of patients; therefore, the available data were insufficient for a systematic longitudinal evaluation of changes in CSF cell count, protein, glucose, and chloride within individual patients.

Finally, because this was a retrospective observational study, the identified associations cannot establish causality. In particular, the higher frequency of antibacterial therapy in the unidentified etiology group should be interpreted as an association with etiological status rather than as a direct causal effect of enterovirus RT-PCR confirmation.

Despite these limitations, the use of standardized clinical and laboratory data, the clear molecular definition of the study groups, and recruitment through the city’s only specialized pediatric infectious diseases center strengthened the consistency and local representativeness of the analysis.

## Figures and Tables

**Table 1:** Age distribution of pediatric patients included in the study.

Age Category	Number (*n*)	Proportion (%)
<1 year	1	1.15
1–4 years	12	13.79
5–9 years	27	31.03
10–14 years	33	37.93
15–17 years	14	16.09
Total	87	100.00

**Table 2:** Time from disease onset to hospital admission and severity-related indicators in children with aseptic meningitis.

Time from Disease Onset to Admission	*n* (%)	Severe Disease, *n* (%)	ICU, *n* (%)	Length of Hospital Stay, Days, Me [Q1; Q3]	CSF Cell Count, Cells/µL, Me [Q1; Q3]
Day 1	13 (14.9)	0 (0.0)	0 (0.0)	9 [8; 10]	34 [25; 71]
Day 2	25 (28.7)	1 (4.0)	1 (4.0)	9 [8; 10]	91 [29; 311]
Day 3	17 (19.5)	0 (0.0)	0 (0.0)	8 [7; 9]	115.5 [51.25; 201.75]
Days 4–5	21 (24.1)	0 (0.0)	0 (0.0)	10 [8; 10]	159 [48.5; 248]
Day ≥ 6	11 (12.6)	1 (9.1)	1 (9.1)	9 [7.5; 9]	145 [46.5; 542.5]
Total	87 (100.0)	2 (2.3)	2 (2.3)	9 [8; 10]	92 [34; 287.5]

**Table 3:** Monthly distribution of hospitalizations among children with aseptic meningitis.

Month	Number of Cases (*n*)	Proportion (%)
January	1	1.15
February	4	4.60
March	5	5.75
April	1	1.15
May	2	2.30
June	23	26.44
July	32	36.78
August	18	20.69
September	1	1.15
Total	87	100.00

**Table 4:** Peripheral blood inflammatory parameters at admission according to etiological group.

Indicator	EV+, Me [Q1; Q3]	Unidentified Etiology, Me [Q1; Q3]
White blood cell count (WBC, ×10^9^/L)	7.35 [6.00; 9.98]	10.70 [8.10; 13.00]
Absolute neutrophil count (ANC, cells/µL)	5476 [3905; 7603]	8849 [5812; 11,247]
Neutrophils (%)	74.00 [65.00; 81.00]	85.00 [78.25; 87.00]
Lymphocytes (%)	23.00 [17.00; 30.00]	11.50 [10.00; 19.25]

**Table 5:** Cerebrospinal fluid parameters at admission according to etiological group.

Indicator	EV+, Me [Q1; Q3]	Unidentified Etiology, Me [Q1; Q3]
CSF cell count (cells/µL)	92.0 [39.0; 290.0]	92.0 [26.0; 248.0]
CSF protein (g/L)	0.220 [0.200; 0.280]	0.230 [0.210; 0.275]
CSF glucose (mmol/L)	3.30 [3.00; 3.50]	3.30 [3.20; 3.60]
CSF chloride (mmol/L)	119.0 [118.0; 120.0]	119.0 [118.0; 120.0]

**Table 6:** Correlations between CSF parameters in EV+ aseptic meningitis.

Pair of CSF Parameters	Spearman’s ρ	*p*-Value
CSF cell count–CSF protein	0.710	<0.0001
CSF cell count–CSF glucose	−0.030	0.8354
CSF cell count–CSF chloride	−0.034	0.8081
CSF protein–CSF glucose	−0.001	0.9937
CSF protein–CSF chloride	−0.089	0.5282
CSF glucose–CSF chloride	0.341	0.0134

**Table 7:** Correlations between CSF parameters in the unidentified etiology group.

Pair of CSF Parameters	Spearman’s ρ	*p*-Value
CSF cell count–CSF protein	0.619	0.0003
CSF cell count–CSF glucose	−0.373	0.0461
CSF cell count–CSF chloride	−0.324	0.0861
CSF protein–CSF glucose	−0.371	0.0478
CSF protein–CSF chloride	−0.265	0.1643
CSF glucose–CSF chloride	0.317	0.0933

**Table 8:** Nuchal rigidity according to time from disease onset to admission.

Time from Disease Onset to Admission	Clearly Positive Nuchal Rigidity, *n* (%)	Equivocal or Absent Nuchal Rigidity, *n* (%)	Total, *n*
Day 1	5 (38.5)	8 (61.5)	13
Day ≥ 2	42 (56.8)	32 (43.2)	74
Total	47 (54.0)	40 (46.0)	87

**Table 9:** Antibacterial therapy according to etiological group.

Etiological Group	Antibacterial Therapy, Yes, *n* (%)	Antibacterial Therapy, No, *n* (%)	Total, *n*
Enterovirus-positive, PCR-confirmed	15 (26.3)	43 (73.7)	58
Unidentified etiology	15 (51.7)	14 (48.3)	29
Total	30 (34.9)	57 (65.1)	87

## Data Availability

The data supporting the findings of this study are not publicly available due to privacy and ethical restrictions related to the use of pediatric medical records. Data may be made available from the corresponding author upon reasonable request and subject to institutional and ethical approval.

## Abbreviations

The following abbreviations are used in this manuscript:

ANC	Absolute neutrophil count
CI	Confidence interval
CNS	Central nervous system
CSF	Cerebrospinal fluid
EV+	Enterovirus-positive
HSV-1/2	Herpes simplex virus types 1 and 2
ICU	Intensive care unit
OR	Odds ratio
PCR	Polymerase chain reaction
WBC	White blood cell count
